# Introductory Address*Address delivered at the opening of the Veterinary Department, University of Pennsylvania, October 2d, 1884.

**Published:** 1885-01

**Authors:** Rush Shippen Huidekoper

**Affiliations:** Dean of the Veterinary Faculty, University of Pennsylvania


### THE JOURNAL

OF

### COMPARATIVE MEDICINE AND SURGERY.

VOL. VI. JANUARY, 1885. No. 1.

### ORIGINAL COMMUNICATIONS.

### Art. I.—INTRODUCTORY ADDRESS.*

BY RUSH SHIPPEN HUIDEKOPER, M.D., V.S.

*Dean of the Veterinary Faculty, University of Pennsylvania.*

Mr. Provost and Gentlemen :

I have, to-day, the honor to deliver the first address of this new Department of the University of Pennsylvania for the instruction of Veterinary Medicine.

I feel that I have a right to the pleasure and pride I take in the position which you have awarded me, as a Pennsylvanian, an alumnus of this University, and as a member of the family of the founder of its medical department which, for one hundred and nineteen years, has stood at the head of medical teaching in the United States ; but I am awed by the responsibility which it places upon me. At the founding of the medical department the country was new; and any advance given to the people for their education was a boon which they welcomed, no matter how small it was. The physician and surgeon were so needed that they rose rapidly to a position which was socially better than it had been in the Mother country ; every addition

* Address delivered at the opening of the Veterinary Department, University of Pennsylvania, October 2d, 1884.

to the ranks of medicine was regarded as a public benefaction, for life is always man's greatest care.

In founding a Veterinary school, we have much labor to perform. While a few people fortunately look upon the philanthropic side of Veterinary medicine, the majority only employ a Veterinary surgeon as a means of saving or of utilizing so many dollars and cents in the form of a domestic animal. Popular prejudice has classed the "Horse Doctor" and the "Cow Leech" with the most ignorant farrier, and has tainted him with the reputation for dishonesty of the proverbial horse dealer; medical men have classed him with the least educated empiric of their own cast, and, with the exception of a few individuals, no one has thought for a moment that the responsibility of the average ignorance was upon himself and his government.

Happily there are always men enterprising beyond their fellows, and throughout the country are many practitioners who have had the diligence to labor and the intelligence to appreciate what their experience has shown them: the want of journals and the small demand for, and high price of Veterinary books, except of the "Universal Stock Book and Veterinary Compendium" order, has prevented these men from being known outside of their own locality. Medical publishers hesitate to print the work of a Veterinary Surgeon unless it is of unexceptionable merit, and books of technical worth are only bought by the few physicians whose personal tastes interest them in animals as a pastime or for laboratory research; books which are within the scope of the layman's understanding are of little accuracy and value and are apt to condemn the author in the eyes of the scientific man.

In establishing an institution for the advancement of Veterinary knowledge, and in asking intelligent and reputable men to select it for a profession whereby they may gain a reputation and livelihood, we have to contend with the prejudice which ignorance has attached to Veterinary Surgeons as a class, and with the reluctance which the aspirants for this title feel, in offering to devote a long period of hard work to gain that which their neighbor, the farrier, acquired the day he opened a suppurating corn in a lame horse and sent it home sound, or the cow-leech took to himself when he gave some chance herb to a cow down with milk fever and she recovered, as they sometimes do, with the aid of nature. Before entering upon the causes which led to the foundation of this department of the University and its aims for the future, I will give a short review of the development of Veterinary Medicine in other countries and in our own.

VETERINARY MEDICINE

derives its name from the Latin "*Veterinarium*," "*Veterinaria*," Veterinary Medicine, "*Veterinarius*," a Veterinary Surgeon, are terms coming from the "*Veterinae*" or "*Veheterinae*," the general term used by the Romans for beasts of burden or pack animals, from "*vehere*," to carry. Lenglet, however, claims that the term is of much older and of Celtic origin, being derived from "*vee*," or "*vieh*," cattle, and the verb "*teeren*," to be sick. Nearly all languages employ words from this same root; French, "*Veterinaire*," Italian, "*Veterinaria*," South Germany, Hungarian and Russian, "*Veterinär*," but in North Germany "*Thierarzt*" and "*Thier-medicin*" (animal doctor and animal medicine) are more generally used.

Veterinary Medicine comprises not only the study of Anatomy, Physiology, Chemistry, Materia Medica and diseases in their relationship to animals, but includes with equal importance the laws of breeding animals, and of raising and training them to be of greatest service to man, whether as motors or as machines for the production of milk, food and clothing; these uses in turn necessitate a knowledge of farriery and the inspection of meat when used as food.

The earliest references to the diseases of animals are found contemporaneously with the first medical writings. Æsculapius in mythological history includes a knowledge of horses, which he derived from Chiron, the Centaur. Hippocrates (460-377 B. C.) described the symptoms of diseases and the remedies to be used in animals. Zend Avesta, the Arab, and Charaka, the Hindoo, mention several of their maladies. Ebers shows that dissection was carried on during the earliest Egyptian dynasties, and probably much more frequently in animals than in man on account of the rigid, religious rules in regard to the dead. The Egyptians had as many specialties as a medical school of to-day, and distinct mention is made of doctors for fowls. Numerous references are made in the Bible to diseases of animals, and to herbs used in curing their troubles, and in the Mosaic laws we have clear orders for the inspection of meat and the division of animals into the pure and the impure. Diocles (360 B. C.) derived most of his knowledge of Anatomy from animals, and Xenophon (445 B. C.), in his treatise on cavalry, describes some of the ailments of horses and especially speaks of founder. Aristotle (384-322 B. C.) wrote a work of some size on animal medicine which has been translated with great care by Doctors Aubert and Wimmer. Pamphylus of Alexandria (200 B. C.), Florentius, and Magon of Carthage at about the same date wrote works which were complete for the time ; the latter was translated by Dionysius of Utica.

In early times the practice of animal medicine was almost exclusively confined to the shepherds and farriers, who rarely raised themselves above the common ignorance of the day. The surgical operations were limited to castration of the males of all species, and to the castration of female swine, which was also done by the earliest nomad races, and at the beginning of the Christian era was a well-known operation in Italy and Gaul. In the prime of the Grecian rule of the world, we find accounts of doctors for horses, who had, however, but a summary knowledge of diseases and blemishes, but who kept in accord with the spirit of the age in proposing great numbers and varieties of curative medicines. The writings of these men, composed almost entirely of letters, were collected in the tenth century by order of the Greek emperor, Constantine Porphyrogennetors, and were printed in the sixteenth century in both Greek and Latin text. The Roman emperors employed Veterinary surgeons in their armies, and the Emperor Augustus ordered the erection of hospitals for sick animals styled "Veterinarium" in contradistinction to the Valetudinarium or hospital for sick soldiers. The Roman writers on agriculture described numerous diseases of cattle, sheep, goats and swine, but their works are only of historical interest. Cato (234-149 B. C.), Varro (116 B. C.), in his *De re Rustica*, and Celsus (40 B. C.), are full of the superstitions of the age, and ascribe to the stars, to the moon and the various natural phenomena the greatest influence on diseases of animals and the remedies to be used in healing them. Columella (40 A. D.), in volumes six and seven of his twelve volumes "*De re Rustica*," treats extensively of animals. He recommends bleeding, describes castration and the use of the hot iron, and splints for fracture. Pedanius Dioscoridius, twenty years later, gives an account of hydrophobia. Pliny, the younger, and Galen speak of the scab in sheep. In the middle of the fourth century Apsyrtus established the diagnosis of strangles from glanders, gave a description of moon blindness, founder, tetanus, cough, tuberculosis and several of the contagious diseases, and he proposed curative means of extreme common sense. At the latter end of the same century Vegetius Renatus, a Latin, collected most of the known writings of his predecessors, and compiled a work of Veterinary Medicine. From the seventh to the thirteenth centuries we find scarcely a trace of literature on veterinary subjects except from the pens of Abu Bekr, Avicenna (980-1037 A. D.), and Ibu el Beilhar (1248 A. D.); all of them Arabs. Then as now among the Arabs the art of healing a horse was regarded as a gift of God, belonging to special families and transmitted by them to their descendants. To offer them pay would be an insult, and their only reward is the most profuse hospitality from their neighbors.

During the early days of the Middle Ages the rulers of States were with a few exceptions too much occupied with wars to devote any thought to the advancement of science or to the promotion of agriculture. Under Frederick II., however, Jordanus Ruffus (1194 to 1250 A. D.) wrote a book of considerable value, in which spavin is described among other blemishes with great credit. In 1270 Theodoric, Bishop of Cervia, also wrote a book of value. The superstition of the Greek and Roman days, which perverted the symptoms of diseases and rendered all study of a rational nature futile, was in the Middle Ages replaced by a superstition more deleterious still to the advancement of any knowledge of animal diseases. The epidemics of the contagious diseases were considered a visitation and punishment of God, and it was thought improper to treat such sick beasts or to dissect their dead bodies. During the fifteenth century a school of cavalry was established in Naples and from it developed men with considerable veterinary attainments. Carraccioli, Grisone and others have left us books in great numbers.

The sixteenth century is the real commencement of a practical Veterinary era. At this time the wars of Europe were at perfection, the warriors had learned to protect their horses with heavy armor and required good horses to carry it; the gentler amusement of tournaments was indulged in alike by warriors and the secular and ecclesiastical princes and nobles, and required horses of spirit and speed to satisfy their ambition; hawking, other sports and the advancing refinement of civilization, which brought ladies, priests, scientific men and the artist followers in the amusements and travels of the courts, demanded palfreys and hinnies for their use. All this led to the breeding of better animals and produced numerous writers concerning the raising and care of the horse; his diseases and blemishes, the mode of curing them and equitation. The breeds of horses at this period in Italy had attained such a reputation that popes, cardinals, princes and all the greater nobles had their special brands and marks, and most of the books contained cuts of them with a description of the peculiar merits of the animals.

In 1590 the Senator Carlo Ruini, of Bologna, a celebrated teacher of medicine in several of the North Italian Universities, published a large and valuable work in folio on the anatomy and blemishes of the horse. He founded in the University at Bologna the first, and to-day the greatest, veterinary museum in the world. In the seventeenth and eighteenth centuries horses had acquired a relatively high value, and the numerous works which appeared on Hippology contained additional chapters on the diseases of the animals. Some attention was, at this, the so-called "stable-master's period," paid to dogs, hawks and other sporting animals; officers of the army and officials of the breeding studs were obliged to apply themselves to the cure of their ailments. In the eighteenth century the Rinderpest ravaged over the most of Europe; princes and governments commissioned the celebrities in medicine of the day to search for a remedy for the treatment or prevention of this pernicious disease. Several of the governments recognized the necessity for institutions for veterinary studies, but the jealousies of the stud masters on one hand and the military and epidemic authorities on the other, prevented the accomplishment of any definite plan.

In 1762, Claude Bourgelat, a French advocate, who was a lover of horses and as an amateur had attained considerable knowledge of animals and of medicine, placed the fruits of his labor and his extraordinary intellect to use. He founded from his own resources, which were limited, a school for teaching Veterinary medicine, at Lyons, in the centre of France; the only qualification demanded from scholars was a good character; the course extended over one year and treated principally of the horse. The success and fame of this school was immediate and great; not only were a large number of French scholars attracted to it, but most of the neighboring governments sent students to learn the merits of it. The French Government, which has always been the foster mother of science, now assumed the responsibility of the institution, enlarged it, and in 1765 called Bourgelat to Paris to establish a second school. This was placed at Alfort, on the site of the Royal Menagerie, and special attention was paid to cattle and sheep. There was at this time in Paris a private school rich in the teaching of Lafosse, the younger, but from unfortunate personal and political differences between Lafosse and Bourgelat no fusion of their teaching could ever be accomplished.

While Austria, Prussia and the greater German States, England, Denmark and Italy, resolved at once to profit by the example of France, the realization of their plans differed greatly and was not everywhere immediately completed. Italy and the Teuton races were the first to follow with success; their institutions were started under two distinct plans; one the founding of a complete Veterinary faculty, the other the addition of a single Veterinary chair to the existing Universities.

The University plan suffered from being unable to furnish sufficient clinical material, enough instruction in anatomy, and because the teaching was allied too closely to that of human medicine, too little practical instruction being given in the elementary parts of proper veterinary training and in the study of animal epidemics.

The coeducation of veterinary and medical students was strongly urged in a memoir of Cothenius, the body physician of Frederick the Great; he also argued that medical students should have a knowledge of animal epidemics so that they might afterwards officiate as Veterinary Inspectors. The first veterinary school to follow those in France was at Turin, in Piedmont, under Charles Emanuel III, King of Sardinia, in 1769; that at Copenhagen, Denmark, was founded in 1773 with Abildgaard in charge.

In Vienna, Austria, an advanced farrier's school, where a few operations were performed, existed from 1767 to 1777; Vienna had long been renowned for its guild of farriers, of whom a monument stands to-day in the Graben in the form of a tree stump converted into a column of iron by the horse-shoe nails which each smith drove into it on becoming a member of the guild. In 1764 the Empress Maria Theresa sent a soldier named Scotti, an apothecary named Mengman, and a certain Haller, to Lyons, for two years, who upon their return gave a limited course of instruction with success. In 1769 the Empress sent a surgeon named Wolstein accompanied by a smith to Alfort, where they remained two years, taking advantage also of Lafosse's clinic in Paris; they then travelled through England, Holland, Denmark, and Germany, and returned in 1775, having spent six years in preparing themselves for their work. This complete training entitled Wolstein to the consideration he received at the hands of the Emperor, who granted him his demands for a course of two years embracing anatomy, exterior anatomy, dietetics, breeding, shoeing, practice of medicine, materia medica, botany and chemistry; also a stable for thirty horses, six to eight cows and swine, and fifteen to twenty smaller animals. The result of this foundation has been the permanent establishment of one of the most methodical schools of Europe. The teachers for other schools were mostly recruited from surgeons and smiths who were sent by their Governments to the French schools, and a number of institutions were rapidly founded; in Hanover 1778, Dresden 1780, Milan 1787, Berlin and Munich 1790, London 1791, Madrid 1793, Giessen 1798, Petersburg (Russia) 1808, Naples 1815, Berne 1816, Zurich 1819, Skara in Sweden, Stuttgart and Utrecht 1821, Edinburgh and Toulouse 1825, Alexandria in Egypt, and Lisbon 1830, Cureghem near Brussels 1832, Warsaw 1840, Constantinople 1842, and others.

In England the Royal College of Veterinary Surgeons was founded in 1791 by a number of noblemen and rich cattle owners at the instigation of a French veterinarian, Vial de Saint Bel, who became the first director. It never received government aid, but has been supported by subscriptions of members of the society, by scholars' fees, by an annual subscription from the Agricultural Society and by the board of Animals in its Hospital. Two institutions of a similar nature have been undertaken in recent years in London by Ainslie and Gamgee, but they were short lived, though the latter did much to awaken an interest in scientific training. The Dick College in Edinburgh, Scotland, founded in 1825, has furnished many teachers and practitioners of renown, and by recent rich bequests promises to take on renewed vigor. The New School in Edinburgh under Professor Williams has just been re-established in fine buildings. Glasgow has also a school. In Italy the schools of Turin, Milan and Naples draw the largest number of students, while there are also institutions rich in their museums, libraries and laboratories at Bologna, Pisa, Parma and Modena, with secondary schools at Perugia and several smaller towns. The teachers are all government officers.

In Germany many of the veterinary chairs in the Universities have disappeared, or are little known; but that of Giessen has always held a well deserved reputation, and in Halle, Professor Putz is making a name for his chair through his valuable scientific work on the contagious diseases. The Berlin school is the great clinical school of Germany, while Dresden and Munich are well-known for their work in anatomy and laboratory research.

In addition to the schools of Warsaw and Petersburg in Russia, others have been founded in Cracow, Dorpat in Livonia, and in the Kassan; these schools are attached to the medical and surgical faculties and require five years' study. The course is most thorough, and from each graduating class are selected by competitive examination two students, who, under pay and at the expense of the government, are sent to the other schools of Europe to perfect themselves in special branches; on their return they pass an examination which if satisfactory attaches them to the faculty, which they enter when a vacancy occurs. The professional and military standing of veterinary surgeons in Russia is probably the highest attained in any country. In Italy the graduates rank as doctors and the diploma is a university degree. In Austria the faculty must possess both the veterinary diploma and that of Doctor of Medicine. In France and Germany the faculties are recruited from among veterinary surgeons, but while the military veterinary surgeons enter as officers in France, they only attain that grade in Germany on becoming senior veterinary surgeon of a regiment, but it must be remembered that military surgeons in Prussia were non-commissioned officers in 1840. In Holland the faculty was composed entirely of medical men until 1851, when veterinary surgeons were admitted as teachers. In England the position of a veterinary surgeon has much improved in recent years; while the military veterinarian became an officer in the early part of the century, the position of the practitioner kept steadily in the background of the medical man, who in turn remained an apothecary until the medical profession was entered by men with titles to their names. It has been mainly due to the efforts of Mr. Fleming, the chief of the Veterinary Department of the Army, that a great improvement has taken place in the profession, and the last ostracism was removed in June, 1883, when he obtained for the Military Veterinary Surgeon the entrée to court.

The course of study in the European schools, while everywhere thorough, varies considerably in its details. It is shortest in England, where three partial years only are demanded. These are devoted essentially to making practitioners, and during the long vacations the students are supposed to be serving with preceptors. For many years English instruction was too exclusively devoted to the horse, but recently much attention has been paid to cattle and other animals, and laboratories for practical teaching are being added, which promise a greater amount of scientific medical education. The *Veterinarian*, a monthly journal, was established in 1828, and has continued uninterruptedly since ; there are many veterinary books in English, but unfortunately too many of them are of a routine character and better suited to the stableman than to the medical man. There are, however, numerous exceptions, and the names of Bracy Clark, Percivall, Williams and Fleming and others will always be honored. French, German and Italian books are comparatively limited in numbers, but are of scientific value. The oldest of veterinary journals, the *Receuil de Médecine Veterinaire*, was established in 1824, and the oldest German journal, the *Vierteljahrschrift für Wissenschaftliche Veterinärkunde*, in Vienna, in 1851. The Austrian and Hungarian institutions teach for three years, with a two years' course for higher grade farriers. In these there is much more laboratory work. The magnificent new institution at Buda Pesth has just been built on ample ground and fitted with every facility for theoretical and practical work. At its side the Agricultural School is in course of construction, and many of the chairs will be common to both. The German schools teach for three and a half years, while the Belgian, Italian and French cover four years in their course of study. The school at Alfort, near Paris, is par excellence the great clinical school, where a hundred animals can be seen each day. Berlin, Lyons and Vienna have large clinics and do more laboratory work. The Toulouse and Swiss schools, with that at Utrecht, have the greatest reputation for cattle practice, and at Munich special attention is given to diseases of the eye in the lower animals, and for this branch a journal is now published.

While there have often been individual veterinary surgeons well known outside of their own profession, it has been within very recent years that we can count, with pride, enough scientific men to show a marked elevation in the standing of our colleagues. But recently we have lost Ercolani, of Bologna, who was known throughout the scientific world for his researches in comparative anatomy, histology of the organs and animal parasites; greater, perhaps, to an Italian was his reputation as a patriot and a statesman in aiding the consolidation of Italy. Gurlt, also, was a veterinary teacher; Thiernesse, the late Director of the Cureghem School, was Secretary of the Academy of Medicine in Brussels; Bouley, to-day Vice-President of the Académie des Sciences in Paris, and Professor at the side of Milne-Edwards, in the Museum, was the greatest veterinary clinician ever known. Chauveau, the anatomist and physiologist, is Director of the Veterinary School in Lyons, and Professor in the Medical Faculty, in which positions he preferred to remain when he refused the chair of Claude Bernard; Bollinger, Siedemgrotszky, Heusinger, Goubaux and others whose names are well known in scientific journals, are veterinary surgeons. In the staff of assistants who accompanied Pasteur to Egypt to study the cholera was Hocard, a veterinary teacher in Alfort.

While preparing myself for my position here, I had the opportunity of visiting many of the schools of which I have just spoken, and of working in several of them, and I beg to be allowed this occasion to publicly testify my thanks and gratitude for the almost universal courtesy, politeness and aid which I received. It was first shown me as your representative, although in many cases it developed into warm personal friendships, and you will allow me to especially mention M. Bouley, M. Goubaux and the Faculty of Alfort, M. Chauveau and the Faculty of Lyons, M. Marey, the Faculty of the Vienna School, Professors Dieckerhoff, at Berlin, and Leisering, at Dresden, Lanzillotti Buonsanti, at Milan, and Mr. Fleming and Professor Williams, in England and Scotland. To the memory of Ercolani, I can only add the feeling of reverence which every one had who knew him personally.

IN AMERICA

the advance in Veterinary Medicine has been far from keeping pace with our national reputation for energy and self-preservation.

In 1806, Dr. Benjamin Rush, of this University, who had just been in Europe and had seen the success of the institutions then a few decades old, wrote a letter to the Agricultural Society of Philadelphia, and urged the importance of adding a Veterinary Department to the University. He called attention to the agricultural prospects of the country, and his letter was discussed before the Society in 1807, but nothing practical was done. Our domestic animals have steadily increased in numbers and in value since that time. We had in 1852—horses, 5,000,000; cattle, 17,000,000; sheep, 22,000,000; swine, 30,000,000; value, $600,000,000, and to-day we have in the United States, horses, 10,838,111—value, $765,041,308; mules, 1,871,079—value, $148,732,390; milch cows, 13,125,685—value, $396,575,405; oxen, etc., 28,046,477—value, $611,549,109; total cattle, 41,171, 762—value, $1,008,124,314; sheep, 49,237,-

291—value, $124,365,335; hogs, 43,270,086—value, $291,951,-221. Total, 176,488,329 animals—value, $2,338,215,268, and there are perhaps in the United States only some 500 Veterinary Surgeons who hold certificates showing that they are properly qualified to practice. An importation of the Russian Rinderpest into the port of New York would probably be followed by the destruction of 13,000,000 cattle, or $300,000,000 worth of property in twelve months.

Valuable breeds of animals have been imported and developed here until individual horses, cows and sheep, reach the enormous value of thousands of dollars. With the increase in the number of animals the ordinary accidental and sporadic diseases have of course increased in the same ratio, but with the augmented number of valuable animals wanted here and there over the country for breeding purposes, with the increased number of horses sent to the large cities for motors, and the thousands of cattle shipped for food in dirty, non-disinfected cars and boats, the increase of traumatic and contagious diseases has been in much greater proportion. Our Government has not done the first thing toward furnishing men capable of combating these scourges; even in the army the handful of veterinary surgeons are not recognized as officers, and have so little authority that, on an outbreak of glanders, they have not the power to condemn or sequestrate an animal if the Colonel thinks it has the disease in what his ignorance calls a non-contagious stage. The still smaller number of officers appointed by the Treasury Department to establish quarantine in order to protect us from imported diseases, has been composed of competent men, but too few in numbers, and without sufficient means and law at their disposal to take proper precautions. The contagious diseases are left for the States to cope with alone, each protecting itself as it sees fit, regardless of its neighbor.

The first veterinary record made in the United States was in Philadelphia, 1818, when we find in the registry of the clerk of the Eastern District that "James Carver hath deposited in this office the title of a book the right whereof he claims as author in the words following: 'The Farriers' Magazine or Archives of Veterinary Science, containing the Anatomy, Physiology and Pathology of the Horse ad other Dome tic

Animals.'" Nine years later, John Rose, a Prussian graduate, settled in New York; about the same year the well-known Mr. Michener began practice in Pennsylvania. In 1851, a Mr. G. H. Dadd, a self-named Veterinary Surgeon, started a Veterinary Journal in Boston which lived about a year, to be revived in 1855 as the *American Veterinary Journal*, and the same year Mr. Dadd and several associates formed the first veterinary school in the country, which, however, soon disappeared.

The New York College of Veterinary Surgeons was chartered in 1857, and up to 1875 led a feeble existence; during this time it issued some eighteen diplomas. The Pennsylvania College, chartered in 1866, has continued its organization, but without a regular course of instruction. Two years later the Illinois Industrial University and Cornell added Dr. Prentice and Professor James Law to their faculties, and have given regular lectures. They were followed by Amherst (1869), the Ohio Agricultural College (1870), and Ames, Iowa. In 1875 the American Veterinary College was formed in New York from the New York College of Veterinary Surgeons, with Professor Liautard, a French graduate at its head. This school has steadily increased in value and number of students. It was the first medical school in the State of New York to require a matriculation examination. It has issued diplomas to 133 of its students, many of whom are in Pennsylvania. In 1862 a school was established in Toronto which, like the New York school, demands but two years' study. The Montreal school, founded in 1866, and directed by Mr. MacEachran, requires three winters. Recently a new school has been established in Chicago, and one at Minneapolis. These schools are dependent on scholars' fees for support, and with the precedents of the medical schools of the country, find that two winters are all that can with pecuniary profit be demanded for forcing into the heads of young men, often with but little previous education, the elements of medicine, with its vast amount of practical details, and the long row of diseases with their variations, in half a dozen widely different species of animals—less time than it takes for a shoemaker's apprentice to learn to cobble shoes, a clerk to become a book-keeper, or a farrier's boy to be trusted to put ordinary shoes on a horse. Harvard University established a Veterinary Department last year, and has a hospital of some size. It requires three years' study, and gives a thorough course of instruction. The school is in charge of Professor Lyman. The United States Veterinary Medical Association, formed in 1863, is composed of the leading veterinary surgeons throughout the country, and has held semi-annual meetings at New York and Boston until the present autumn, when the meeting was held in Cincinnati. In 1870 the late Dr. S. D. Gross, with his keen appreciation and ready heart for the demands of medical education, framed and presented the following resolutions to the American Medical Association, but his noble attempt to advance our profession failed—" was lost by a large majority."

" *Whereas*, We regard the cultivation of Veterinary Science of the most vital importance, not only to the advancement of human medicine, but also for reasons of political economy and agricultural interest;

" *Resolved*, First.—That we recommend the State and County Medical Societies to use their influence in the establishment and support of veterinary schools in their respective States.

" Second.—That they ask the Governors of their respective States to recommend in their messages to their Legislatures the importance of establishing veterinary colleges, and that appropriations be made to support them.

" Third.—That they recommend the Governor and the State Legislature, when organizing Boards of Health, to appoint one or more thoroughly educated Veterinary Surgeons to be associated as commissioners with other medical officers.

*Resolved*, That we recommend the employment of Veterinary Surgeons in the army, and one in the Agricultural Department, with rank and pay of other medical officers."

Our Veterinary Department has been contemplated for some time, and was rendered practicable through the acquisition by the University of this piece of land from the city of Philadelphia, and the liberality of Mr. J. B. Lippincott and Mr. Joseph E. Gillingham, who have furnished the means for these substantial buildings and outfit. Unfortunately a veterinary school cannot be ordered and completed like a primary schoolhouse, and we have but the cornerstone of what I believe will be a great institution.

We open to-day with the veterinary course of the first year only; our matriculants, twenty in number, have been required to show a certificate of sufficient previous education, or have passed a preliminary examination equivalent to that of the Medical Department. This requirement is too little for men who should be qualified to undertake the mathematics needed in a problem of chemical analysis, electricity, or the value of a muscular movement in the physiological study of an animal, or to handle easily the technical terms derived from Greek and Latin which medicine has found it proper to employ for nomenclature, but it is sufficient to guarantee that the student has enough education to appreciate what will be taught him, with great diligence and labor on his own part.

Our students will learn this year on the same footing as those of the Medical Department, the study of chemistry, with its practical courses, under Professor Wormley; they will follow the course of Materia Medica and Pharmacy under Dr. Miller and Dr. Alexander Glass, V.S., in order to familiarize them with the specialties required in compounding veterinary medicines; they will have the full course of Physiology from Professor Allen, and where this is inadequate for veterinary instruction, as it is necessarily prepared for the students of human medicine, there will be supplementary lectures by Professor Smith, who will also direct them in practical work with special reference to the domestic animals; the elementary course in general Pathology under Professor Tyson, will be the same for the veterinary and the medical students; Professor Rothrock will not only give them general Botany, but will pay special attention to the plants used for forage and their nutrient value; Professor Parker, in his course of Zoology, after giving them the general laws of the development and classification of animals, will dwell upon the helminths and animal parasites; the course of Anatomy will embrace the horse, cow, sheep, goat, hog, dogs, cats and poultry, and in the course of Histology the tissues of these animals will be used; from the microscope the student will go to the blacksmith's shop, where he will learn to forge and to shoe the horse's foot. This last course has never been practically carried out in the English-speaking schools, but is essential to the veterinary surgeon. He will never be a farrier, and in practice will avoid even taking off a shoe when he can get any one else to do it for him; but shoeing is the cause of nine-tenths of the surgical evils in the horse, and without a thorough practical knowledge of it, it is impossible to obtain from or show a blacksmith what one wants.

In the second year, medical or organic Chemistry will be taught and examinations will be held, the course of Physiology, Botany, Zoölogy and Anatomy will be finished, the students will commence their lectures in therapeutics, with practical demonstrations of the effects of drugs on the domestic animals. They will continue the course of general Pathology. With the second year will commence the lectures on Surgical Pathology, Internal Pathology and the contagious diseases or practice of medicine. These same lectures will be continued the third year with the addition of lectures on obstetrics and zoötechnics, or the laws of breeding and raising animals, and the modes employed for obtaining the greatest use from them, as they may be destined for animal motors or machines for the production of milk, wool or flesh. A course will be given on the preparation of butcher meat, showing the most humane methods of slaughtering animals for food, the preparation of the meat and the signs of unhealthy or diseased flesh in the living or dressed animal. There will also be lectures on Sanitary Police, familiarizing students with the inadequate laws of this country and those of other countries, which they may use as models when called upon to consult in these matters. With the commencement of the third year the student will enter the hospital, and during it and the following year will have direct charge of the sick animals. They will keep the clinical records, administer the medicines, perform minor operations, and in case of death, make the autopsies. Each in turn will serve in the hospital pharmacy and prepare all medicines required in the institution.

Such, gentlemen, is the plan of study which we have laid out for making veterinary surgeons. This training, thoroughly carried out, will give us men fully capable of being the scientific peers of any Doctor of Medicine. It will form men completely fitted for any trust in an animal epidemic or in the minor details of a routine practice. A man with this education will hold his head up among his fellows, and when a stableman calls for a "horse doctor" will feel that he is called out as a respected professional man to do good, and that he is beyond the suspicion of being asked to use his knowledge to share in a deception or a fraud.

For the instruction of the first year we are as fully equipped as a school can be at its beginning, with but a small museum, and the rough edges of the various parts of this educational machine still unworn.

For the second and third years we are not yet prepared, and we depend upon your generosity and that of your neighbors to complete this department. We have here such a piece of ground as is unobtainable in any other large city in the United States, and if we take advantage of it before it is appropriated to other needs, we will have an establishment equal to any in Europe. We need stables for at least fifty sick horses at once, which should be built with the prospect of enlarging in the future. In this hospital we will take sick animals to board, and it in part will be self-supporting; but there will be many animals with diseases tedious to treat, which will be abandoned by their owners, and we need a fund for the support of such cases. There are many cases of disease among the horses of poor carters, which are readily curable, but the owner cannot afford the fees of a Veterinary Surgeon, nor the expense of an animal standing idle and eating the food which its work should be paying for; these are the cases which furnish many of the examples of misery that the Society for the Prevention of Cruelty to Animals is called upon to alleviate; in many cases the driver is not naturally brutal, but at home there are wife and children to be supported, and the suffering beast is the best he can afford. We need a fund for the support of such animals, which will be judiciously sent us by the Agents of the Society for the Prevention of Cruelty to Animals and by the practicing Veterinary Surgeons of the town. Such a fund will be a double charity, the suffering animal will be relieved, and the knowledge that it can be done without depriving a poor owner of his daily bread will induce the latter to be more charitable himself. We need a cattle dairy of at least fifty cows. This should be largely self-supporting after once established; it will enable us to teach the students practical obstetrics and many of the details of cattle practice of which the usual veterinary graduate is absolutely ignorant; we need dormitories for the students where those from away can be comfortably lodged and learn their Alma Mater is their home for the time. For our veterinary students who are to give personal supervision to the animals in the hospital, it is essential; for the entire university, it is a necessity. Harvard, Yale or Princeton Alumni meet each other like Free Masons, and though otherwise strangers, they become rapidly intimate over the past, present and future of their college, because they roomed in the same building, messed at the same table, and had the same associations; this feeling is absent in the Alumni of the University of Pennsylvania, except, perhaps, among the medical graduates, who had their small cliques in their boarding houses. Dormitories will do much to make the university popular, they will tie the graduate's memories to his student life and induce him to take an interest in the future of the institution. We need a Botanical Garden, and with ample frontage for all our buildings on the side of this triangle, a beautiful spot is left in the centre for its construction; there exists already a small fund for this purpose left by the late Dr. George B. Wood. We need endowments for the Chairs. The Chair of Surgery must be filled before the next year, and we should be untramelled in our choice and be able to select the best talent without being influenced by the pecuniary value of so many students.

In fine, gentlemen, we need, and I am sure we will have, your support for this undertaking.

